# An Improved Fuzzy Connectedness Method for Automatic Three-Dimensional Liver Vessel Segmentation in CT Images

**DOI:** 10.1155/2018/2376317

**Published:** 2018-10-29

**Authors:** Rui Zhang, Zhuhuang Zhou, Weiwei Wu, Chung-Chih Lin, Po-Hsiang Tsui, Shuicai Wu

**Affiliations:** ^1^College of Life Science and Bioengineering, Beijing University of Technology, Beijing 100124, China; ^2^College of Biomedical Engineering, Capital Medical University, Beijing 100069, China; ^3^Department of Computer Science and Information Engineering, Chang Gung University, Taoyuan 33302, Taiwan; ^4^Department of Medical Imaging and Radiological Sciences, College of Medicine, Chang Gung University, Taoyuan 33302, Taiwan; ^5^Department of Medical Imaging and Intervention, Chang Gung Memorial Hospital at Linkou, Taoyuan 33302, Taiwan; ^6^Medical Imaging Research Center, Institute for Radiological Research, Chang Gung University and Chang Gung Memorial Hospital at Linkou, Taoyuan 33302, Taiwan

## Abstract

In this paper, an improved fuzzy connectedness (FC) method was proposed for automatic three-dimensional (3D) liver vessel segmentation in computed tomography (CT) images. The vessel-enhanced image (i.e., vesselness image) was incorporated into the fuzzy affinity function of FC, rather than the intensity image used by traditional FC. An improved vesselness filter was proposed by incorporating adaptive sigmoid filtering and a background-suppressing item. The fuzzy scene of FC was automatically initialized by using the Otsu segmentation algorithm and one single seed generated adaptively, while traditional FC required multiple seeds. The improved FC method was evaluated on 40 cases of clinical CT volumetric images from the 3Dircadb (*n*=20) and Sliver07 (*n*=20) datasets. Experimental results showed that the proposed liver vessel segmentation strategy could achieve better segmentation performance than traditional FC, region growing, and threshold level set. Average accuracy, sensitivity, specificity, and Dice coefficient of the improved FC method were, respectively, (96.4 ± 1.1)%, (73.7 ± 7.6)%, (97.4 ± 1.3)%, and (67.3 ± 5.7)% for the 3Dircadb dataset and (96.8 ± 0.6)%, (89.1 ± 6.8)%, (97.6 ± 1.1)%, and (71.4 ± 7.6)% for the Sliver07 dataset. It was concluded that the improved FC may be used as a new method for automatic 3D segmentation of liver vessel from CT images.

## 1. Introduction

Hepatocellular carcinoma (HCC) is one of the most common malignancies in the world, especially in China with the fifth morbidity and the third mortality [1, 2]. Nowadays, main treatments to HCC include surgical resection, liver transplantation, and local thermal ablation [3]. Treatment planning and navigation based on medical imaging are essential for these procedures. Among different medical imaging modalities, computed tomography (CT) is commonly used for the guidance of liver tumor treatment. Three-dimensional (3D) segmentation of liver vessel is critical in CT-based liver tumor treatment planning and navigation. However, manual segmentation of liver vessel in CT images is time consuming and tedious. Thus, there is a demand for computerized 3D segmentation of liver vessel in CT images [4, 5].

Currently, computerized liver vessel segmentation techniques can be classified into region growing [6–8], active contour models or level sets [9], graph cuts [10–12], extreme learning [13], deep learning [14], and fuzzy logic [15, 16]. However, it is still challenging to extract liver vessel in CT images, especially in those with low contrast [4]. Region growing methods [6–8] are simple with low computational cost, but they are sensitive to seed point location and threshold. Active contour models or level sets [9] are among mainstream vessel segmentation algorithms, but they have limitations of complex initialization and typically use speed function which implicitly assumes that images are of good contrast. Graph cuts [10–12] are segmentation methods based on graph theory, but they are partly limited by the shrinking bias problem. Machine learning methods [13, 14] can take into account the diversity of liver vessel topologies and features, but they generally require plenty of training samples or long training time. Fuzzy connectedness (FC) methods [15, 16] are based on fuzzy logic. FC describes spatial connectedness between each voxel, rather than just focusing on intensity. Recently, Guo et al. [15] and Wang et al. [16] have demonstrated the potential of FC in liver vessel segmentation. However, for FC-based liver vessel segmentation in CT images, there are still issues to be addressed, including unsatisfying segmentation accuracy (especially for low-contrast CT images), requirement on multiple seeds, and sensitivity to initialization.

In this paper, an improved FC method was proposed for automatic 3D liver vessel segmentation in CT images. The vessel-enhanced image (i.e., vesselness image) was incorporated into the fuzzy affinity function of FC, rather than the intensity image used by traditional FC. An improved vesselness filter was also proposed based on the Jerman's vesselness filter [17] introduced recently. The fuzzy scene of FC was initialized by using the Otsu segmentation algorithm, and the quantity of seeds required was reduced to one which was generated automatically. The proposed method was evaluated on 40 cases of clinical CT image volumes, including low-contrast images. Experimental results demonstrate that the improved FC method can overcome the drawbacks of traditional FC and yield more satisfying segmentation performance.

## 2. Materials and Methods

**Figure 1:**
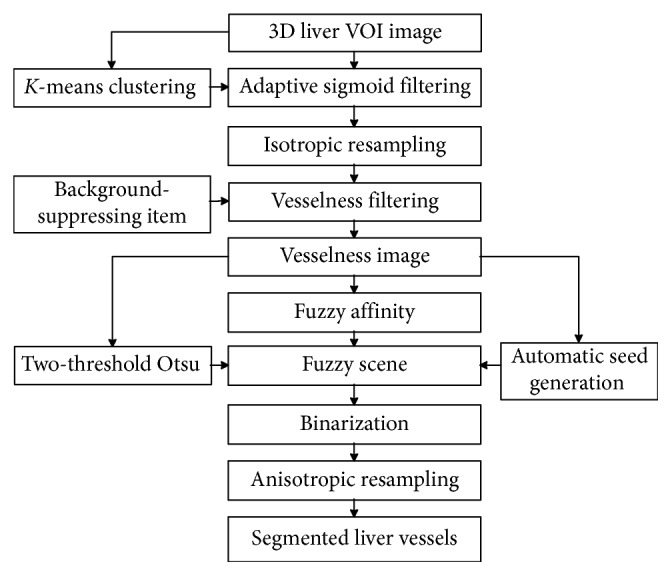
Flowchart of the improved fuzzy connectedness method for automatic 3D segmentation of liver vessel from CT images.

Figure 1 shows the flow chart of the improved FC method. First, the liver volume of interest (VOI) image was obtained by using the liver mask, which could be obtained by using liver segmentation approaches [18]. The liver VOI image was then contrast enhanced by an adaptive sigmoid filtering which was initialized by *K*-means clustering and isotropically resampled. Subsequently, the improved vesselness filter was used to enhance the liver vessel and suppress the background, and a 3D vesselness image was obtained. Then, a 3D fuzzy scene was constructed with the 3D vesselness image by (1) incorporating the improved vesselness into the fuzzy affinity function of FC, (2) initializing the fuzzy scene by the Otsu algorithm, and (3) generating automatically one single seed. Finally, the 3D liver vessel was segmented on the basis of the 3D fuzzy scene and anisotropically resampled.

### 2.1. Dataset

Both simulated data (*n*=60) and clinical CT data (*n*=40) were used. The synthetic dataset VascuSynth [19, 20] was provided by the Medical Image Analysis Lab, School of Computing Science, Simon Fraser University, Canada. VascuSynth contains 10 groups of data, which are publically available at http://vascusynth.cs.sfu.ca. Each group consists of 12 randomly generated images with different quantity of bifurcations. Six groups of data were randomly selected; among them, the images with bifurcations ≥11 were included in this study. Gaussian white noise was also added to the raw data. The level of the Gaussian white noise was indicated by *σ*^2^, the variance of the noise. In this study, Gaussian white noise with *σ*^2^ = 30, 45, and 60 were added.

The clinical CT image datasets, 3Dircadb and Sliver07, were used. 3Dircadb contains 20 cases of contrast-enhanced CT (CE-CT) images. 3Dircadb was provided by the Research Institute against Digestive Cancer, France, and is publically available at http://www.ircad.fr/research/3dircadb. The pixel spacing is 0.56–0.86 mm, and the slice thickness is 1–4 mm. The number of slices ranges from 64 to 502, and the in-plane resolution is 512 × 512 pixels. The gold standard of liver vessel was provided by 3Dircadb, which was manually delineated by radiologists. Sliver07 contains 30 cases of CE-CT images, including 20 training sets and 10 testing sets. The 20 cases of training data are publically available at http://www.sliver07.org and were included in this study. However, Sliver07 did not provide the gold standard of liver vessel. Therefore, radiologists were asked to manually delineate the liver vessel to serve as the gold standard for the 20 cases of training data of Sliver07. The number of slices, in-plane resolution, and interslice resolution range from 64 to 394, from 0.58 to 0.81 mm, and from 0.7 to 5.0 mm, respectively.

### 2.2. Improved Vesselness Filter

The multiscale Hessian matrix-based filter (vesselness filter) is commonly used for vessel enhancement [4, 17]. Classical vesselness filters were proposed by Sato et al. [21] and Frangi et al. [22]. Since then, Li et al. [23], Erdt et al. [24], and Xiao et al. [25] proposed improved methods for enhancing the vasculature. Recently, Jerman et al. [17] proposed a novel vesselness filter and demonstrated that it outperformed traditional vesselness filters. For completeness, the Jerman's filter was introduced briefly as below. Let *λ*_*i*_, *i*=1,2,3 denotes the Hessian eigenvalues of a 3D image at each coordinate **x**. Considering the ideal eigenvalues' relationship *λ*_2_ ≈ *λ*_3_ ∧ |*λ*_2,3_| > >|*λ*_1_| in vasculature, Jerman et al. [17] constructed a novel Hessian eigenvalues function to improve the enhancement performance by using a two-step piecewise compensation. In CT images, the magnitudes of *λ*_2_ and *λ*_3_ were lower at the vascular boundary or in the low-scale vessel (|*λ*_3_| ≥ |*λ*_2_| ≈ |*λ*_1_|⟶*Low*), which did not match the ideal Hessian eigenvalues relationship in vasculature, resulting in significant attenuation of the vesselness response. Therefore, Jerman et al. [17] performed a piecewise compensation on the eigenvalue *λ*_3_:

(1) $$\begin{array}{c} \lambda_{\rho }\left(\sigma \right)=\left\{\begin{array}{cc} \lambda_{3}, & \text{if }\lambda_{3}>\tau \;\max_{x}\lambda_{3}\left(x,\sigma \right),\\ \tau \;\max_{x}\lambda_{3}\left(x,\sigma \right), & \text{if }0<\lambda_{3}\leq \tau \;\max_{x}\lambda_{3}\left(x,\sigma \right),\\ 0, & \text{otherwise,} \end{array}\right. \end{array}$$

where *σ* is the vessel scale and *τ* is a threshold between 0 and 1. In addition, traditional vesselness filters would suppress blob-like structures and obtain poor response at vascular nodes (|*λ*_1_| ≈ |*λ*_2_| ≈ |*λ*_3_|⟶*High*). Thus, Jerman et al. [17] compensated the ellipsoid structure conforming to the condition *λ*_2_ ≥ *λ*_*ρ*_/2 > 0 to construct the final vesselness function:

(2) $$\begin{array}{c} \upsilon_{k}=\left\{\begin{array}{cc} 0, & \text{if }\lambda_{2}\leq 0\;\vee \;\lambda_{\rho }\leq 0,\\ 1, & \text{if }\lambda_{2}\geq \lambda_{\rho }/2>0,\\ \lambda_{2}^{2}\left(\lambda_{\rho }-\lambda_{2}\right){\left[3/\left(\lambda_{2}+\lambda_{\rho }\right)\right]}^{3}, & \text{otherwise}. \end{array}\right. \end{array}$$

Jerman et al. [17] evaluated their method on clinical image datasets of lung, cerebral, and fundus vasculatures. However, for the task of liver vessel enhancement in CT images, the Jerman's filter would enhance the liver contour, liver parenchyma, and noise. Therefore, the Jerman's filter was improved by incorporating adaptive sigmoid filter for contrast enhancement and by incorporating a background-suppressing item into the vesselness function of the Jerman's filter (Equation (2)).

The adaptive sigmoid filter is defined as

(3) $$\begin{array}{c} I_{\text{sigmoid}}={\left(1+\exp\left(-\frac{I_{\text{VOI}}-\beta }{\alpha }\right)\right)}^{-1}, \end{array}$$

where *I*_sigmoid_ is the filtered image, *I*_VOI_ is the liver VOI image, and *β* and *α* represented the intensity center and the intensity range of the vasculature. In this study, *β* and *α* were obtained adaptively by the *K*-means clustering (*K* = 5). The internal structure of *I*_VOI_ was clustered into five regions with corresponding cluster centers. With the value of the intensity centers ranking from low to high, the five regions corresponded to the background, liver tumor, liver parenchyma, low-intensity vessel mixed with liver parenchyma, and high-intensity vessel, respectively. With the intensity means of the last two regions (*m*_1_ and *m*_2_), parameters *β* and *α* are calculated by

(4) $$\begin{array}{c} \left\{\begin{array}{c} \alpha =\left(m_{2}-m_{1}\right)/2,\\ \beta =\left(m_{2}+m_{1}\right)/2. \end{array}\right. \end{array}$$

The background-suppressing item, 1 − *e*^−*R*_*s*_^2^/2*γ*^, was incorporated into Equation (2), yielding

(5) $$\begin{array}{c} \upsilon =\left\{\begin{array}{cc} 0, & \text{if }\lambda_{2}\leq 0\;\vee \;\lambda_{\rho }\leq 0,\\ 1, & \text{if }\lambda_{2}\geq \lambda_{\rho }/2>0,\\ \lambda_{2}^{2}\left(\lambda_{\rho }-\lambda_{2}\right){\left[3/\left(\lambda_{2}+\lambda_{\rho }\right)\right]}^{3}\left(1-e^{-R_{s}^{2}/2\gamma }\right), & \text{otherwise,} \end{array}\right. \end{array}$$

where $R_{s}=\sqrt{\lambda_{1}^{2}+\lambda_{2}^{2}+\lambda_{\rho }^{2}}$ and *γ* is the background suppression coefficient, which was optimally set at *λ*_*ρ*_/3.

Finally, the vesselness response was combined by calculating the maximum response of *υ* in each scale *σ*, *σ* ∈ [*σ*_min_, *σ*_max_]:

(6) $$\begin{array}{c} I_{\text{vesselness}}=\sup\left\{\upsilon :\sigma_{\min}\leq \sigma \leq \sigma_{\max}\right\}, \end{array}$$

where *I*_vesselness_ is the final vessel-enhanced image (vesselness image). The improved vesselness filter algorithm is summarized in Algorithm 1.

### 2.3. Improved Fuzzy Connectedness

FC involved three kinds of fuzzy relationships: fuzzy adjacency, fuzzy affinity, and fuzzy connectivity. Fuzzy affinity represented the local similarity of the voxel pair (*c*, *d*) in the entire image scene *C*, denoted by *μ*_*κ*_(*c*, *d*) ∈ [0,1]:

(7) $$\begin{array}{c} \mu_{\kappa }\left(c,d\right)=\mu_{\alpha }\left(c,d\right)\left[\omega_{1}h_{1}\left(f\left(c\right),f\left(d\right)\right)+\omega_{2}h_{2}\left(f\left(c\right),f\left(d\right)\right)\right], \end{array}$$

where *μ*_*α*_(*c*, *d*) is the fuzzy adjacency (a monotonic increasing function), and *h*_1_ and *h*_2_ are computed by

(8) $$\begin{array}{c} h_{1}\left(f\left(c\right),f\left(d\right)\right)=e^{-1/2{\left[f\left(c\right)+f\left(d\right)/2-m/s\right]}^{2}},\\ h_{2}\left(f\left(c\right),f\left(d\right)\right)=e^{-1/2{\left[\left|f\left(c\right)-f\left(d\right)\right|-m/s\right]}^{2}}, \end{array}$$

where *f*(·) is the intensity of voxels; *m* and *s* are mean and standard deviation of *f*(·) in the VOI, respectively; and *ω*_1_ and *ω*_2_ are weight parameters, *ω*_1_+*ω*_2_=1.

In this paper, the vesselness image obtained by using the improved vesselness filter was used as the input of the fuzzy affinity function, rather than the intensity image used by traditional FC. The improved fuzzy affinity function, *μ*_*κ*_′(*c*, *d*), is defined as

(9) $$\begin{array}{c} \mu_{\kappa }^{\prime }\left(c,d\right)=\mu_{\alpha }\left(c,d\right)\left[\omega_{1}h_{1}\left(I_{\text{vesselness}}\left(c\right),I_{\text{vesselness}}\left(d\right)\right)+\;\omega_{2}h_{2}\left(I_{\text{vesselness}}\left(c\right),I_{\text{vesselness}}\left(d\right)\right)\right]. \end{array}$$

To adaptively set parameters *m* and *s*, the Otsu segmentation algorithm was adopted to the vesselness image. Two-threshold Otsu was used to yield a binary liver vessel mask. Parameters *m* and *s* are, respectively, set at the mean and standard deviation of the vesselness voxels belonging to the foreground of the vessel mask.

The weight parameters *ω*_1_ and *ω*_2_ are adaptively selected by using the method proposed by Pednekar et al. [26]:

(10) $$\begin{array}{c} \omega_{1}=\frac{h_{1}}{h_{1}+h_{2}},\\ \omega_{2}=1-\omega_{1}. \end{array}$$

**Figure 2:**
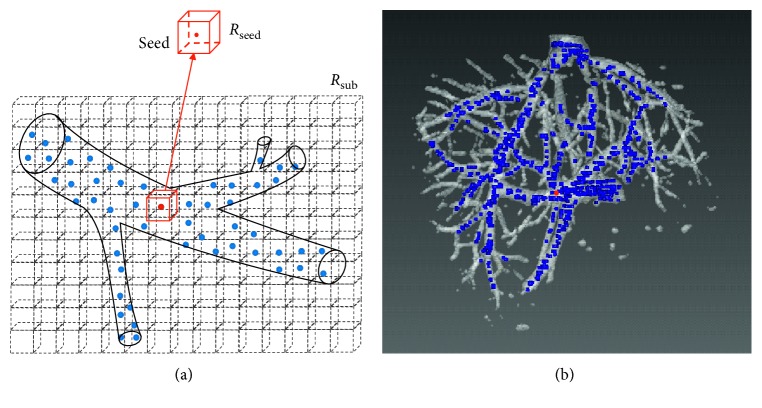
Multiple potential seeds and one single seed generated automatically on the vesselness image. (a) Illustration for automatic seed selection. (b) Multiple potential seeds (blue) and one single seed (red) indicated on the vesselness image.

The fuzzy scene of liver vessel was initialized with one single seed generated automatically on the vesselness image, binarized by a threshold *T*, and anisotropically resampled to yield the final liver vessel segmentation.  Figure 2 illustrates automatic selection of one single seed. In Figure 2(a), the 3D image *I*_vesselness_ was divided into several regions *R*_sub_ of 5 ∗ 5 ∗ 3 voxels. The maximum vesselness voxels at each *R*_sub_ region were selected as potential seeds (denoted by the blue points in Figure 2(b)). Then, the regions of 5 ∗ 5 ∗ 3 voxels around the potential seeds were constructed and denoted as *R*_seed_, with each potential seed being the center of each *R*_seed_ region. The mean of the vesselness of each *R*_seed_ region was calculated. The potential seed having the largest vesselness mean in its *R*_seed_ region was automatically selected as the final single seed, which was indicated by the red point in Figure 2(b). The improved FC algorithm is summarized in Algorithm 2.

### 2.4. Evaluation

To analyze quantitatively the performance of the proposed vessel segmentation method, evaluation metrics including accuracy, sensitivity, specificity, and Dice coefficient were used:

(11) $$\begin{array}{c} \text{Accuracy}=\frac{\text{TP}+\text{TN}}{\text{TP}+\text{FN}+\text{TN}+\text{FP}},\\ \text{Sensitivity}=\frac{\text{TP}}{\text{FN}+\text{TP}},\\ \text{Specificity}=\frac{\text{TN}}{\text{TN}+\text{FP}},\\ \text{Dice}=\frac{2\text{TP}}{2\text{TP}+\text{FN}+\text{FP}}, \end{array}$$

where TP and TN are the numbers of voxels correctly segmented as vessel and background (i.e., nonvessel), respectively; FP and FN are the numbers of voxels incorrectly segmented as vessel and background, respectively.

## 3. Results

**Figure 3:**
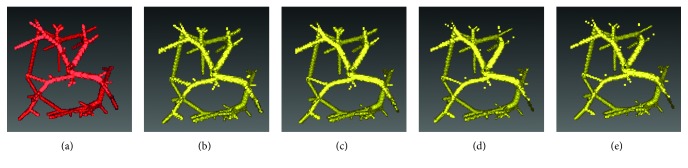
Segmentation of vessel from synthetic data by using the improved fuzzy connectedness method. (a) The gold standard of vessel. (b–e) The vessel segmented from the synthetic data added with Gaussian white noise *σ*^2^ = 0, 30, 45, and 60, respectively.

Figure 3 shows the vessel segmented from the simulated data by using the improved FC method.  Figure 3(a) represents the ground truth; Figure 3(b) shows the segmented vessel on the synthetic data; and Figures 3(c)–3(e) show the segmented vessel on the synthetic data added with Gaussian white noise *σ*^2^ = 30, 45, and 60, respectively. The segmentation performance of the improved FC method on the synthetic dataset (*n*=60) is shown in Table 1, in terms of accuracy, sensitivity, specificity, and Dice coefficient. Although the sensitivity and Dice coefficient were decreased to some extent with increasing the level of Gaussian white noise, the segmentation performance was generally kept stable. It is thus indicated that the improved FC method is insensitive to Gaussian white noise.

**Figure 4:**
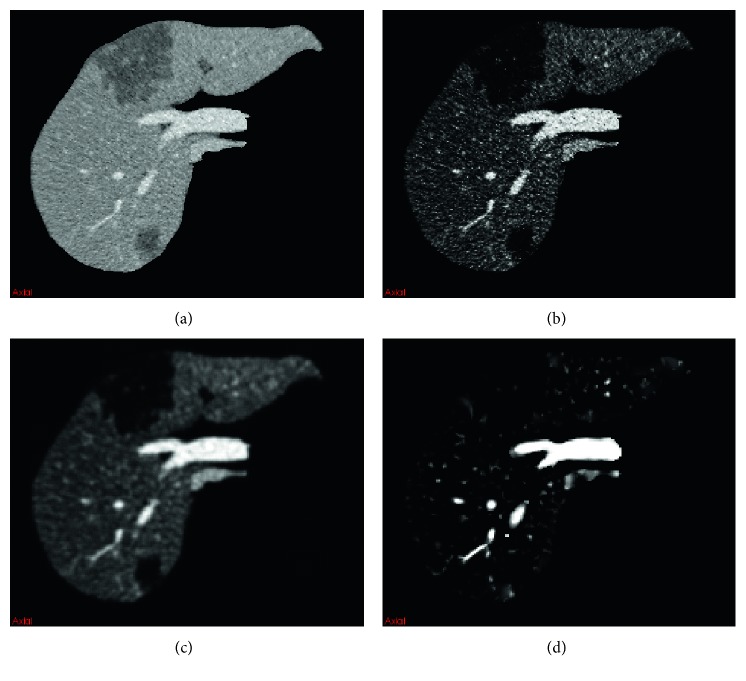
Illustration of the improved vesselness filter. (a) The original CT image. (b) The adaptive sigmoid filtered image of (a). (c) The isotropic resampled image of (b). (d) The improved vesselness filtered image of (c).

**Figure 5:**
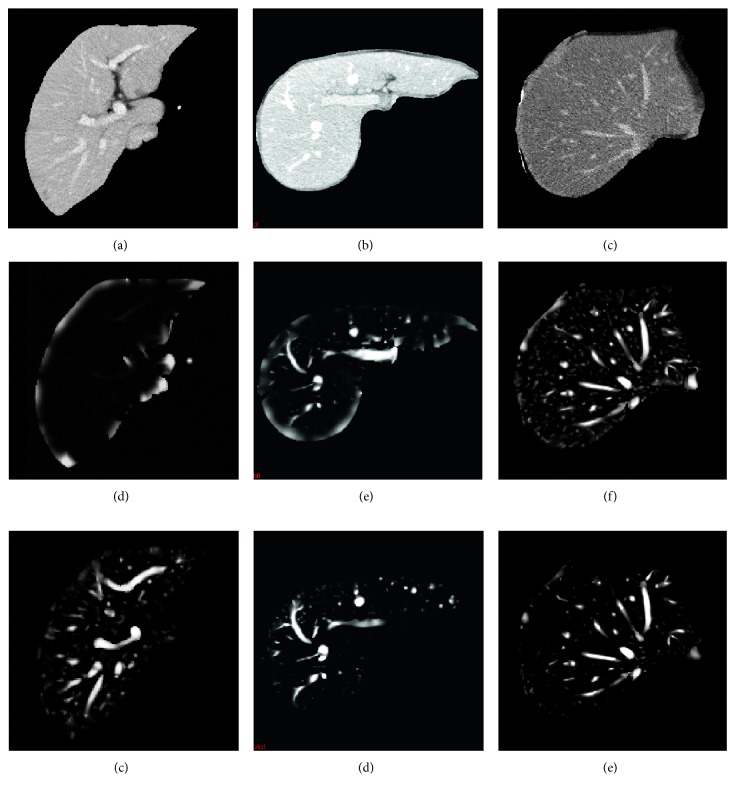
Comparison of the improved vesselness filter with the Jerman's vesselness filter. (a)–(c) The original CT images. (d)–(f) The vesselness images by using the Jerman's vesselness filtering to (a)–(c), respectively. (g)–(i) The vesselness images by using the improved vesselness filtering to (a)–(c), respectively.

Figure 4 shows the vessel-enhanced image by using the improved vesselness filter.  Figure 4(a) shows the original CT image; Figure 4(b) shows the adaptive sigmoid filtered image; Figure 4(c) shows the isotropic resampled image; and Figure 4(d) shows the improved vesselness filtered image. It can be seen that the improved vesselness filter can effectively enhance the vessel while suppressing the background. The vesselness images obtained by using the Jerman's vesselness filter and the improved vesselness filter are shown in Figure 5. The intensity of the vesselness images ranged from 0 to 1. The contrast of vessel in CT images increased from Figures 5(a)to 5(c). Note that the Jerman's vesselness filter enhanced the liver contour and almost neglected the liver vessel for the low-contrast image (Figure 5(d)). As the image contrast increased, there was still undesired enhancement at the liver contour (Figures 5(e) and 5(f)). In addition, the Jerman's vesselness filter could not effectively suppress the background (Figure 5(f)). By contrast, the improved vesselness filter successfully enhanced the vessel while suppressing the background, with little enhancement at the liver contour (Figures 5(g)–5(i)).

**Figure 6:**
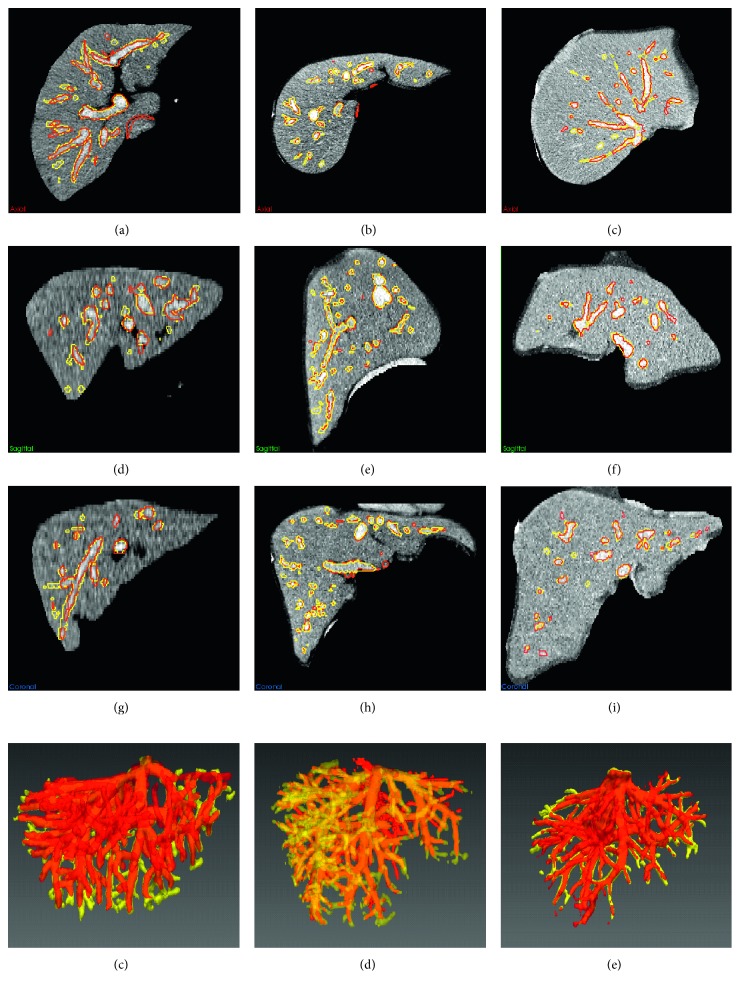
Liver vessel segmented by using the proposed method (yellow). The gold standard of vessel is depicted in red. Each column corresponds to one case. (a)–(c) The axial slices. (d)–(f) The sagittal slices. (g)–(i) The coronal slices. (j)–(k) The 3D view.

**Figure 7:**
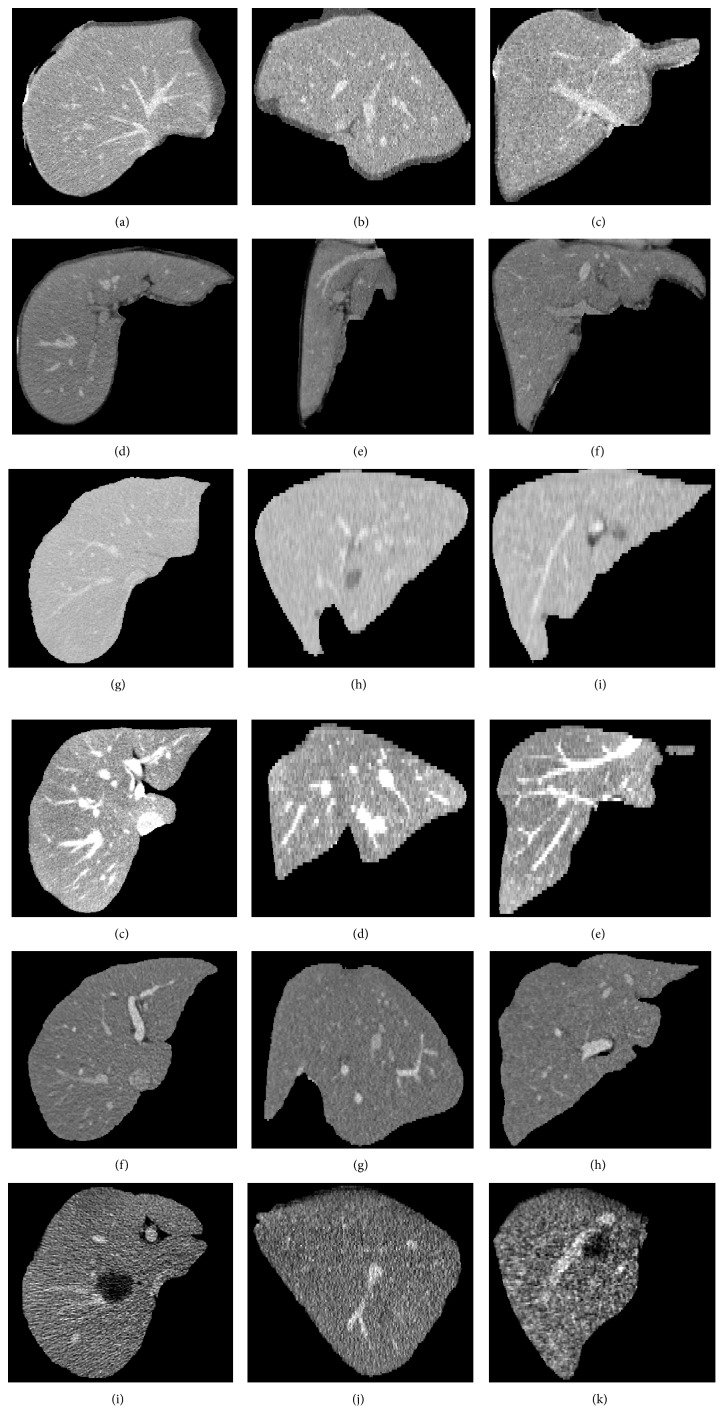
Typical CT images from the 3Dircadb (a)–(i) and Sliver07 (j)–(r) datasets. Each row corresponds to one case. The first, second, and third columns are axial slices, sagittal slices, and coronal slices, respectively. The original CT images in (a)–(i) and (j)–(r) are used in Figures 8 and 9, respectively. For vessel segmentation, (a)–(c) and (j)–(l) are of high contrast, while (d)–(i) and (m)–(r) are of low contrast.

**Figure 8:**
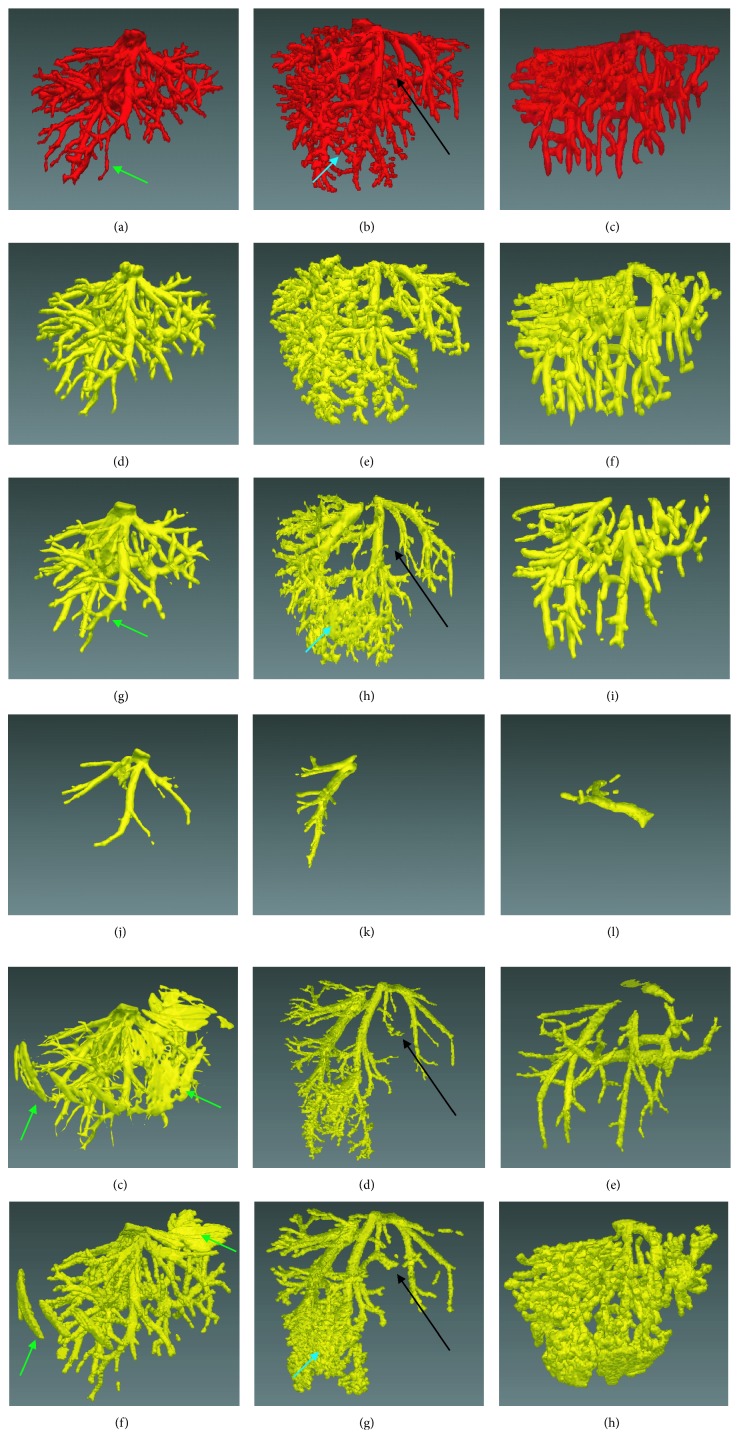
Comparison of the improved fuzzy connectedness (FC) method with traditional segmentation algorithms. Each column corresponds to one case. The original CT images of each case are shown in Figures 7(a)–7(i). (a)–(c) The gold standard of liver vessel. (d)–(f) The vessel segmented by using the improved FC. (g)–(i) The vessel segmented by using traditional FC with multiple potential seeds indicated by the blue points in Figure 2(b). (j)–(l) The vessel segmented by using traditional FC with one single seed indicated by the red point in Figure 2(b). (m)–(o) The vessel segmented by using region growing with multiple potential seeds. (p)–(r) The vessel segmented by using threshold level set with multiple potential seeds.

**Figure 9:**
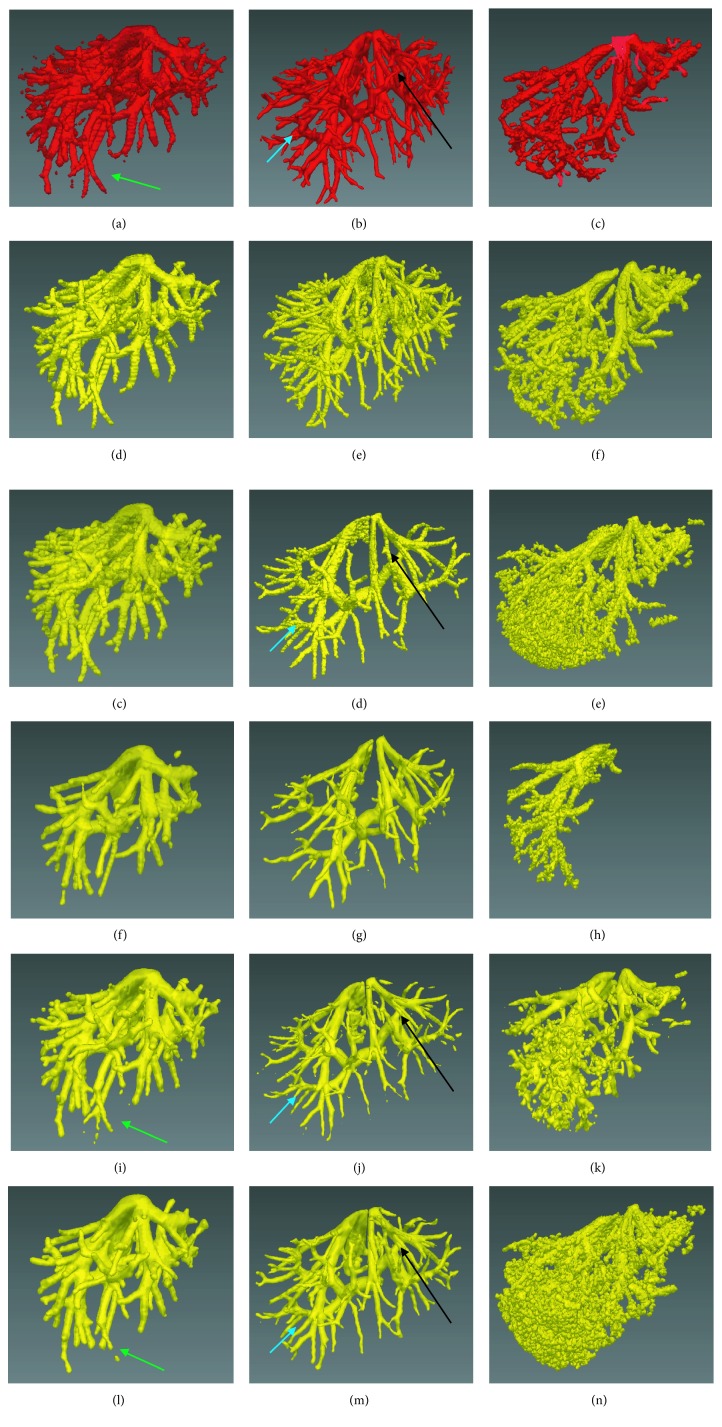
Comparison of the improved fuzzy connectedness (FC) method with traditional segmentation algorithms. Each column corresponds to one case. The original CT images of each case are shown in Figures 7(j)–7(r). (a)–(c) The gold standard of liver vessel. (d)–(f) The vessel segmented by using the improved FC. (g)–(i) The vessel segmented by using traditional FC with multiple potential seeds indicated by the blue points in Figure 2(b). (j)–(l) The vessel segmented by using traditional FC with one single seed indicated by the red point in Figure 2(b). (m)–(o) The vessel segmented by using region growing with multiple potential seeds. (p)–(r) The vessel segmented by using threshold level set with multiple potential seeds.

Figure 6 shows the liver vessel segmented by using the improved FC, depicted by yellow contour or surface. The gold standard of liver vessel is indicated by red contour or surface. The axial slices, sagittal slices, coronal slices, and 3D view are shown in Figures 6(a)–6(c), 6(d)–6(f), 6(g)–6(i), and 6(j)-6(k), respectively. It can be seen that the proposed method yielded satisfying segmentation performance.  Figure 7 shows typical CT images from the 3Dircadb (Figures 7(a)–7(i)) and Sliver07 (Figures 7(j)–7(r)) datasets. The original CT images in Figures 7(a)–7(i) and 7(j)–7(r) are used in Figures 8 and 9, respectively. For vessel segmentation, Figures 7(a)–7(c) and 7(j)–7(l) were of high contrast, while Figures 7(d)–7(i) and 7(m)–7(r) were of low contrast.

Figures 8 and 9 show the comparison of the improved FC method with traditional segmentation algorithms, including traditional FC [27], region growing [27], and threshold level set [27]. Figures 8(a)–8(c) and 9(a)–9(c) show the gold standard of liver vessel. Figures 8(d)–8(f) and 9(d)–9(f) show the vessel segmented by using the improved FC method. Figures 8(g)–8(i) and 9(g)–9(i) show the vessel segmented by using traditional FC with multiple potential seeds indicated by the blue points in Figure 2(b). Figures 8(j)–8(l) and 9(j)–9(l) show the vessel segmented by using threshold level set with multiple potential seeds. Traditional FC with one single seed could not segment completely the liver vessel (Figures 8(j)–8(l) and 9(j)–9(l)). When multiple potential seeds were used for traditional FC, the segmentation performance show the vessel segmented by using traditional FC with one single seed indicated by the red point in Figure 2(b). Figures 8(m)–8(o) and 9(m)–9(o) show vessel segmented by using region growing with multiple potential seeds. Figures 8(p)–8(r) and 9(p)–9(r) shows improved, but it was still unsatisfying (Figures 8(g)–8(i) and 9(g)–9(i)). For region growing and threshold level set with multiple potential seeds, both undersegmentation and oversegmentation of vessel occurred (Figures 8(m)–8(r) and 9(m)–9(r)). It is interesting to discuss the segmentation performance on low-contrast cases shown in Figures 7(d)–7(i) and 7(m)–7(r). If a part of the main vessel was low-contrast, it would be totally unsegmented, as indicated by the black arrows in Figures 8(h), 8(n), 8(q) and 9(h), 9(n), 9(q). When the peripheral vessel was low-contrast, it would be merged (Figures 8(h) and 8(q)) or missed (Figures 9(h), 9(n), 9(d)), as indicated by blue arrows. Even in the high-contrast images shown in Figures 7(a)–7(c) and 7(j)–7(l), part of the vessel was segmented falsely (Figures 8(m) and 8(p)) and the periphery vessel was not segmented (Figures 8(g) and 9(m), 9(p)), as indicated by green arrows. By contrast, the proposed method was capable to segment completely the liver vessel, even for the low-contrast images.

**Figure 10:**
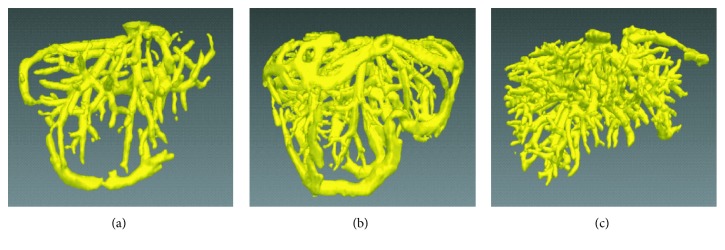
The liver vessel segmented by using the improved fuzzy connectedness method on the basis of the Jerman's vesselness filtering, rather than the improved vesselness filtering. (a) and (b) The Jerman's vesselness filter falsely enhances the liver contour. (c) The Jerman's vesselness filter fails to effectively suppress the background (nonvessel) tissues.

**Figure 11:**
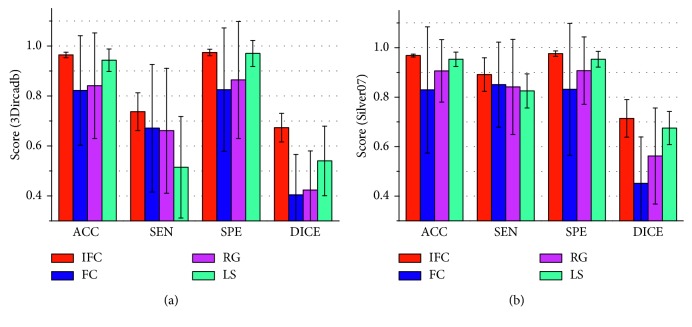
Comparison of the improved fuzzy connectedness (with one single seed) with traditional fuzzy connectedness, region growing, and threshold level set (with multiple potential seeds). (a) and (b) show the accuracy, sensitivity, specificity, and Dice coefficient for the 3Dircadb and Sliver07 datasets, respectively. ACC = accuracy; SEN = sensitivity; SPE = specificity; DICE = Dice coefficient; IFC = improved fuzzy connectedness; FC = fuzzy connectedness; RG = region growing; LS = threshold level set.

To compare further the improved vesselness filter with the Jerman's vesselness filter, the liver vessel segmented by using the improved FC method on the basis of the Jerman's vesselness filtering, rather than the improved vesselness filtering, is shown in Figure 10. Figures 10(a) and 10(b) show that the Jerman's vesselness filter falsely enhanced the liver contour.  Figure 10(c) shows that the Jerman's vesselness filter could not effectively suppress the background (nonvessel) tissues. Quantitative comparison of the improved FC (with one single seed) with traditional FC, region growing, and threshold level set (with multiple seeds) on the 3Dircadb (*n*=20) and Sliver07 (*n*=20) datasets are listed in Table 2 and shown in Figure 11, in terms of accuracy, sensitivity, specificity, and Dice coefficient. It can be observed that the improved FC outperformed traditional FC, region growing, and threshold level set. The average accuracy, sensitivity, specificity, and Dice coefficient of the improved FC method were, respectively, (96.4 ± 1.1)%, (73.7 ± 7.6)%, (97.4 ± 1.3)%, and (67.3 ± 5.7)% for the 3Dircadb dataset and (96.8 ± 0.6)%, (89.1 ± 6.8)%, (97.6 ± 1.1)%, and (71.4 ± 7.6)% for the Sliver07 dataset.

## 4. Discussion

### 4.1. Significance of This Study

3D liver vessel segmentation is critical in computer-assisted liver tumor treatment planning and navigation. FC is an emerging method for image segmentation. However, traditional FC obtained unsatisfying performance for liver vessel segmentation in CT images, and it required multiple seeds and was sensitive to initialization. To address these issues, an improved FC method was proposed in this paper. Our method was fully automatic. The main contributions of this study were as follows. The Jerman's vesselness filter was improved by incorporating adaptive sigmoid filtering and a background-suppressing item. The improved vesselness filter effectively enhanced the vessel and suppressed the background. The improved vesselness response was incorporated into the fuzzy affinity function, increasing the segmentation performance of FC. The fuzzy scene was initialized by two-threshold Otsu with one single seed, reducing the number of seeds and the sensitivity to initialization in traditional FC.

### 4.2. Implementation Details of the Algorithms

The algorithms described in this paper were implemented by using C++ and the Insight Segmentation and Registration Toolkit (ITK) (http://itk.org) [27]. The following ITK classes were mainly used:

1. The improved vesselness filter was implemented on the basis of the class itk::HessianToObjectnessMeasureImageFilter.
2. The improved FC method was implemented on the basis of the class itk::SimpleFuzzyConnectednessScalarImageFilter. This class was also used for the traditional FC segmentation.
3. The *K*-means clustering was implemented by using the class itk::Statistics::ScalarImageKmeansImageFilter.
4. The class itk::SigmoidImageFilter was used for sigmoid filtering.
5. The classes itk::ResampleImageFilter and itk::IdentityTransform were used for isotropic resampling.
6. The class itk::ConfidenceConnectedImageFilter was used for region growing segmentation.
7. The class itk::ThresholdSegmentationLevelSetImageFilter was used for threshold level set segmentation.

Average run time of the proposed algorithm was 200 s for 3Dircadb and 210 s for Sliver07. The improved vesselness filtering took approximately 30 s. The improved FC segmentation also took nearly 30 s. Each of the isotropic resampling and anisotropic resampling took around 60 s.

### 4.3. Sensitivity of the Proposed Algorithm to Key Algorithmic Parameters

**Figure 12:**
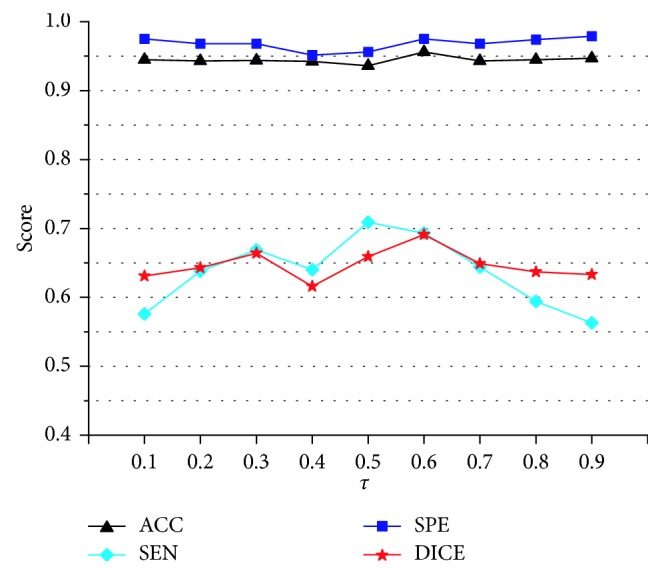
Evaluation of the segmentation performance of the improved fuzzy connectedness method on 10 cases randomly selected from the 3Dircadb dataset for the values of *T* ranging from 0.1 to 0.9 (*T* = 0.05). The value of *T* is optimally set at 0.6. ACC = accuracy; SEN = sensitivity; SPE = specificity; DICE = Dice coefficient.

**Figure 13:**
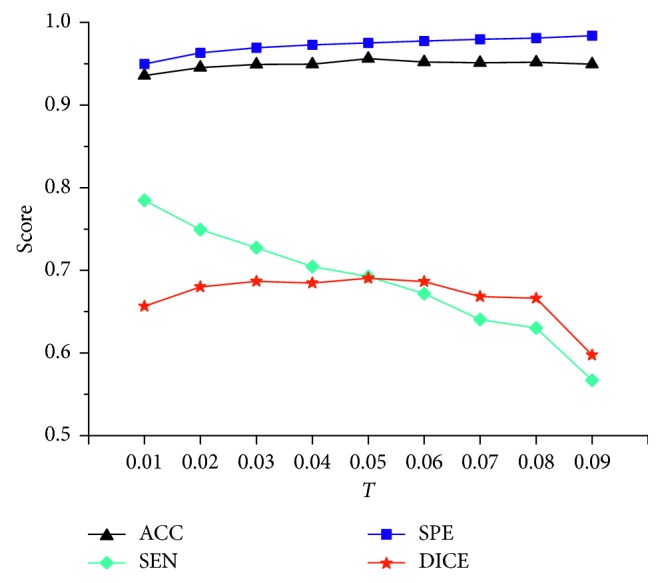
Evaluation of the segmentation performance of the improved fuzzy connectedness method on 10 cases randomly selected from the 3Dircadb dataset for the values of *T* ranging from 0.01 to 0.09 (*T* = 0.6). The value of *T* is optimally set at 0.05. ACC = accuracy; SEN = sensitivity; SPE = specificity; DICE = Dice coefficient.

Sensitivity analysis of key algorithmic parameters in Algorithms 1 and 2 was performed. The vessel scales *σ*_min_ and *σ*_max_ were set on the basis of the findings of Luu et al. [4]. Here, two key algorithmic parameters were analyzed: the threshold *τ* in the improved vesselness filter and the threshold *T* in the improved FC. The threshold *τ* in vesselness filter determined the degree of piecewise compensation on the eigenvalue *λ*_3_. In theory, the smaller the threshold *τ* is, the more enhancement on the vessel boundary would be obtained; however, a too small *τ* is prone to cause undersegmentation. The threshold *T* in FC determined the degree of undersegmentation or oversegmentation. A too small *T* caused undersegmentation, while a too large *T* resulted in oversegmentation. The value of *T* from 0.01 to 0.09 was tested, as the segmented vasculature would be incomplete when *T* > 0.1. For the compromise between undersegmentation and oversegmentation, the value of *T* was firstly fixed to 0.05 to analyze the sensitivity of the proposed algorithm to the threshold *τ*.  Figure 12 shows the average accuracy, sensitivity, specificity, and Dice coefficient of the proposed method on 10 cases randomly selected from the 3Dircadb dataset. The threshold *τ* ranged from 0.1 to 0.9 (*T* = 0.05). The accuracy and Dice coefficient reached peak when *τ* was optimally set at 0.6. Then, the value of *τ* was fixed to 0.6 to analyze the sensitivity of the proposed algorithm to the threshold *T*.  Figure 13 shows the segmentation performance of the proposed method on the 10 randomly selected cases, with *T* ranging from 0.01 to 0.09 (*τ*=0.6). Based on the maximum value of the accuracy and Dice coefficient, the parameter *T* was optimally set at 0.05.

### 4.4. Comparison with Related Work

Table 3 shows a comparison of the improved FC method with related work in terms of segmentation method, dataset, number of cases, automation, precision, and run time. For the run time of the proposed method, it should be noted that each of the improved vesselness filtering and the improved FC segmentation only took around 30 s.

Firstly, the proposed method was compared with related work that used the 20 cases of the Sliver07 training dataset. Oliveira et al. [7] used region growing for liver vessel segmentation, but they only performed visual assessment for the segmentation. Ahmadi et al. [28] segmented liver vessel by using fuzzy *C*-means clustering and initialized the parameters by the genetic algorithm. Though the run time was shorter, the training process was more complex, and the accuracy and specificity of Ahmadi et al. [28] were lower than those of the proposed method. Then, the proposed method was compared with related work that used the 3Dircadb dataset. Huang et al. [14] segmented liver vessel on the 20 cases of 3Dircadb by using the 3D U-Net network. Their method reduced the need for the quantity of training data, but it required long training time (48 h). The accuracy, sensitivity, specificity, and Dice coefficient of Huang et al. [14] were slightly higher than those of the proposed method. Sangsefidi et al. [11] employed graph cuts for segmenting liver vessel, but they evaluated their method on only few cases of 3Dircadb.

Finally, the proposed method was compared with related work that used clinical data other than Sliver07 and 3Dircadb. These studies mostly used CT angiography (CTA) images, which were specific CT for vasculature with clear vascular boundary. However, in the context of computer-assisted liver tumor treatment planning and navigation, CE-CT images may be used more commonly, as liver tumors could be observed in CE-CT images. Though region growing methods had relatively higher operation efficiency, they are depended on the number and distribution of seeds, resulting in unsatisfied segmentation performance even in high-contrast CTA images [4]. Graph cuts and level set methods would take long time to segment liver vessel [9–12]. Esneault et al. [10] just showed the segmentation on one case of data, and they reported that the segmented vascular branches needed to be registered, which would take more time. Zeng et al. [12] reported that their method only achieved good performance on high-contrast CTA images, so their method might be restricted in practical applications when only low-contrast CT images are available. The similar issue existed in Shang et al. [9] and Zeng et al. [13]. Shang et al. [9] evaluated the sensitivity by the number of vascular nodes (denoted as SEN∗ in Table 3), but this evaluation metric may not be rigorous. In comparison with Guo et al. [15] and Wang et al. [16] which increased the time efficiency of traditional FC, this study focused on improving the segmentation performance and reducing the number of seeds and the sensitivity to initialization. In addition, our method did not require manual interaction to select the seed.

### 4.5. Limitations and Future Work

One limitation of this study is the small number of clinical data with the gold standard (40 cases). More clinical data may be used in future (if possible) to further verify the performance of the proposed method. In addition, the algorithmic steps of isotropic resampling and anisotropic resampling are time consuming, each taking around 60 s. This limitation may be overcome in future work.

## 5. Conclusions

An improved FC method was presented for automatic liver vessel segmentation in CT volumetric images. The Jerman's vesselness filter was improved by incorporating adaptive sigmoid filtering and a background-suppressing item. The improved vesselness filter effectively enhanced the liver vessel while suppressing the background. The improved vesselness response was incorporated into the fuzzy affinity function of FC. The fuzzy scene was initialized by two-threshold Otsu with one single seed generated automatically, reducing the number of seeds and the sensitivity to initialization in traditional FC. The improved FC method was evaluated on 40 cases of clinical CT volumetric images. Experimental results showed that the proposed liver vessel segmentation strategy could achieve better segmentation performance than traditional FC, region growing, and threshold level set. It is concluded that the proposed algorithm may be used as a new method for automatic 3D liver vessel segmentation in CT images.

## Figures and Tables

**Algorithm 1:**
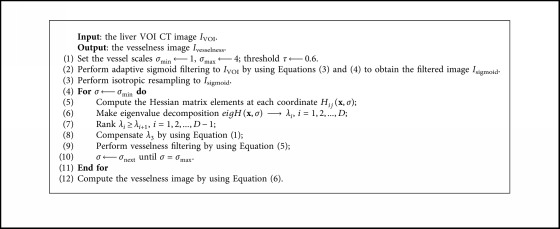
Improved vesselness filter.

**Algorithm 2:**
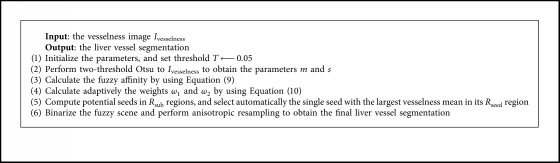
Improved fuzzy connectedness.

**Table 1:** Segmentation performance of the improved fuzzy connectedness method on the synthetic dataset.

	Group 1 (*n*=10)				Group 2 (*n*=10)				Group 3 (*n*=10)				Group 4 (*n*=10)				Group 5 (*n*=10)				Group 6 (*n*=10)			
	ACC (%)	SEN (%)	SPE (%)	DICE (%)	ACC (%)	SEN (%)	SPE (%)	DICE (%)	ACC (%)	SEN (%)	SPE (%)	DICE (%)	ACC (%)	SEN (%)	SPE (%)	DICE (%)	ACC (%)	SEN (%)	SPE (%)	DICE (%)	ACC (%)	SEN (%)	SPE (%)	DICE (%)
*σ* ^2^ = 0	99.7 ± 0.1	96.6 ± 5.1	99.7 ± 0.2	84.3 ± 3.6	99.8 ± 0.1	91.2 ± 4.9	99.8 ± 0.1	86.6 ± 2.6	99.8 ± 0.1	91.5 ± 3.5	99.8 ± 0.1	85.4 ± 3.1	99.8 ± 0.1	91.5 ± 3.5	99.8 ± 0.1	86.0 ± 3.6	99.8 ± 0.1	91.5 ± 4.0	99.9 ± 0.1	85.4 ± 2.4	99.7 ± 0.3	91.0 ± 3.9	99.9 ± 0.1	85.6 ± 3.0
*σ* ^2^ = 30	99.7 ± 0.2	86.7 ± 4.9	99.7 ± 0.2	83.8 ± 4.1	99.7 ± 0.1	87.5 ± 7.3	99.8 ± 0.1	82.2 ± 3.3	99.7 ± 0.1	89.4 ± 4.7	99.8 ± 0.1	80.7 ± 3.1	99.7 ± 0.1	89.9 ± 5.2	99.7 ± 0.1	80.7 ± 3.6	99.7 ± 0.1	90.4 ± 5.2	99.8 ± 0.1	81.0 ± 2.5	99.7 ± 0.1	88.6 ± 6.6	99.8 ± 0.1	80.2 ± 2.5
*σ* ^2^ = 45	99.5 ± 6.3	91.9 ± 4.6	99.7 ± 0.1	81.3 ± 3.0	99.7 ± 0.1	88.3 ± 6.1	99.8 ± 0.1	81.8 ± 4.1	99.7 ± 0.1	90.4 ± 4.5	99.8 ± 0.1	80.7 ± 3.1	99.7 ± 0.1	89.7 ± 5.3	99.8 ± 0.1	80.8 ± 3.5	99.7 ± 0.1	89.1 ± 5.3	99.8 ± 0.1	80.9 ± 2.8	99.7 ± 0.1	87.8 ± 6.6	99.8 ± 0.1	80.6 ± 2.7
*σ* ^2^ = 60	99.6 ± 0.1	91.4 ± 4.3	99.7 ± 0.1	80.6 ± 2.4	99.7 ± 0.1	88.2 ± 5.5	99.8 ± 0.1	82.2 ± 4.1	99.7 ± 0.1	90.3 ± 4.5	99.8 ± 0.1	81.0 ± 3.1	99.7 ± 0.1	90.2 ± 5.1	99.8 ± 0.1	81.1 ± 3.3	99.7 ± 0.1	88.6 ± 5.1	99.8 ± 0.1	81.4 ± 2.6	99.7 ± 0.1	87.2 ± 6.4	99.8 ± 0.1	81.1 ± 2.7

ACC = accuracy; SEN = sensitivity; SPE = specificity; DICE = Dice coefficient.

**Table 2:** Segmentation performance of the improved fuzzy connectedness (with one single seed), traditional fuzzy connectedness, region growing, and threshold level set (with multiple potential seeds).

	Improved fuzzy connectedness				Fuzzy connectedness				Region growing				Threshold level set			
	ACC (%)	SEN (%)	SPE (%)	DICE (%)	ACC (%)	SEN (%)	SPE (%)	DICE (%)	ACC (%)	SEN (%)	SPE (%)	DICE (%)	ACC (%)	SEN (%)	SPE (%)	DICE (%)
3Dircadb (*n*=20)	96.4 ± 1.1	73.7 ± 7.6	97.4 ± 1.3	67.3 ± 5.7	82.2 ± 21.9	67.1 ± 25.5	82.5 ± 24.7	40.4 ± 16.2	84.1 ± 21.1	66.1 ± 25.0	86.4 ± 23.4	42.4 ± 15.6	94.3 ± 4.5	51.5 ± 20.3	97.0 ± 5.2	54.0 ± 13.9
Sliver07 (*n*=20)	96.8 ± 0.6	89.1 ± 6.8	97.6 ± 1.1	71.4 ± 7.6	82.9 ± 25.5	85.0 ± 17.2	83.1 ± 26.7	45.1 ± 18.8	90.6 ± 12.6	84.1 ± 19.2	90.7 ± 13.6	56.2 ± 19.4	95.3 ± 2.9	82.5 ± 6.9	95.3 ± 3.2	67.5 ± 6.7

ACC = accuracy; SEN = sensitivity; SPE = specificity; DICE = Dice coefficient.

**Table 3:** Comparison of the proposed method with related work.

Author	Year	Method	Dataset	#	Automation	Precision (%)	Run time (s)
Oliveira et al. [7]	2011	RG	Sliver07	20	Auto	—	—
Luu et al. [4]	2015	RG	Clinical CTA	51	Auto	ACC = 86.2; SEN = 85.1; SPE = 92.3	—
Esneault et al. [10]	2010	GC	Clinical CTA	1	Auto	—	10–100
Zeng et al. [12]	2017	GC	Clinical CTA	6	Auto	ACC = 97.7; SEN = 79.8; SPE = 98.6	390
Sangsefidi et al. [11]	2018	GC	3Dircadb/Clinical CTA	7	Auto	DICE = 74.0	560
Shang et al. [9]	2011	LS	Clinical CTA	20	Auto	SEN^*∗*^ = 91.0	480
Ahmadi et al. [28]	2016	FCC	Sliver07	20	Auto	ACC = 91.0; SEN = 94.1; SPE = 83.6	27.1
Zeng et al. [13]	2016	ML	Clinical CTA	6	Auto	ACC = 98.1; SEN = 74.2; SPE = 99.3	0.05–0.1
Guo et al. [15]	2015	FC	Clinical CTA	4	Semi	—	112.5
Wang et al. [16]	2016	FC	Clinical CTA	3	Semi	—	22
Huang et al. [14]	2018	DL	3Dircadb	20	Auto	ACC = 97.1; SEN = 74.3; SPE = 98.3; DICE = 67.5	230
Ours	2018	IFC	3Dircadb Sliver07	20 20	Auto Auto	ACC = 96.4; SEN = 73.7; SPE = 97.4; DICE = 67.3 ACC = 96.8; SEN = 84.4; SPE = 97.6; DICE = 71.4	200 210

^*∗*^Evaluation by the number of vascular nodes; CTA = computed tomography angiography; RG = region growing; GC = graph cuts; LS = level set; FCC = fuzzy *C*-means clustering; ML = machine learning; FC = fuzzy connectedness; DL = deep learning; IFC = improved fuzzy connectedness; ACC = accuracy; SEN = sensitivity; SPE = specificity; DICE = Dice coefficient; Auto = automatic; Semi = semiautomatic.

## Data Availability

The VascuSynth dataset is publically available at http://vascusynth.cs.sfu.ca/. The 3Dircadb dataset is publically available at http://www.ircad.fr/research/3dircadb. The training data of the Sliver07 dataset are publically available at http://www.sliver07.org/.
